# Genome Sequence of Listeria monocytogenes 2542, a Serotype 4b Strain from a Cheese-Related Outbreak in Portugal

**DOI:** 10.1128/genomeA.00540-18

**Published:** 2018-06-21

**Authors:** Vânia Ferreira, Rui Magalhães, Gonçalo Almeida, Didier Cabanes, Moritz Fritzenwanker, Trinad Chakraborty, Torsten Hain, Paula Teixeira

**Affiliations:** a CBQF–Centro de Biotecnologia e Química Fina–Laboratório Associado, Escola Superior de Biotecnologia, Universidade Católica Portuguesa, Porto, Portugal; b Group of Molecular Microbiology, IBMC–Institute for Molecular and Cell Biology, i3S–Institute for Research and Innovation in Health, Porto, Portugal; c Institute of Medical Microbiology, German Centre of Infection Research, Site Giessen-Marburg-Langen, Justus-Liebig-University Giessen, Giessen, Germany; d INIAV, IP–National Institute for Agrarian and Veterinary Research, Vairão, Portugal

## Abstract

We report here the draft genome sequence of Listeria monocytogenes 2542, a serotype 4b clinical strain recovered from a placental sample during a cheese-related listeriosis outbreak in Portugal.

## GENOME ANNOUNCEMENT

Listeria monocytogenes, a Gram-positive, short, rod-shaped bacterium, is the causative agent of listeriosis, which is spread through the consumption of contaminated food (1). This pathogen has the ability to cross the intestinal, blood-brain, and fetoplacental barriers; septicemia, central nervous system infections, miscarriages, and stillbirths are the most common forms of this invasive infection (2). The establishment of the disease depends on a number of variables, including the number of ingested bacteria, the pathogenic potential of the strain, and the immunological status of the host (3). A high mortality rate is reported in groups at increased risk for listeriosis, such as pregnant women and individuals with impaired cell-mediated immunity (e.g., HIV/AIDS, cancer, immunosuppressive therapy, and organ transplant) and chronic disease (e.g., diabetes, alcoholism, and liver and renal disease), as well as individuals over 60 years old (4). Although all strains are considered virulent, L. monocytogenes is a highly heterogeneous species. For instance, while there are 13 different serotypes and 4 genetic lineages described for this pathogen, lineage I serotypes 4b and 1/2b are more frequently recovered in cases of human listeriosis (5).

From 2009 to 2012, a listeriosis outbreak linked to the consumption of contaminated cheese occurred in Portugal (6). A high case-fatality rate (36.7%) was reported among the 30 cases of listeriosis identified. The genome sequence of one L. monocytogenes strain isolated from a placental sample of a pregnant woman after stillbirth associated with this epidemic was determined by whole-genome shotgun sequencing (WGS). Chromosomal DNA of L. monocytogenes strain 2542 (serotype 4b) was isolated using a PureLink genomic DNA minikit (Life Technologies). For WGS, a DNA library was created as described in the Nextera XT (Illumina, Inc.) protocol. Quality control of the library was performed using the Agilent 2000 bioanalyzer (Agilent Technologies) and sequenced on the Illumina MiSeq platform using V2 chemistry. Using CLC Genomics Workbench version 6.0, 5,485,526 sequence reads were assembled, resulting in a draft genome of 2,965,111 bp.

### Accession number(s).

This WGS project has been deposited in DDBJ/ENA/GenBank under the accession number FZRK00000000. The version described in this paper is the first version, FZRK01000000.
